# Humoral immunity in dually vaccinated SARS-CoV-2-naïve individuals and in booster-vaccinated COVID-19-convalescent subjects

**DOI:** 10.1007/s15010-022-01817-8

**Published:** 2022-04-11

**Authors:** Vivian Glück, Leonid Tydykov, Anna-Lena Mader, Anne-Sophie Warda, Manuela Bertok, Tanja Weidlich, Christine Gottwald, Josef Köstler, Bernd Salzberger, Ralf Wagner, Michael Koller, André Gessner, Barbara Schmidt, Thomas Glück, David Peterhoff

**Affiliations:** 1 Institute for Clinical Microbiology and Hygiene, University Hospital Regensburg, Regensburg, Germany; 2 Kliniken Südostbayern AG, Klinikum Traunstein, Traunstein, Germany; 3 Department for Infection Control and Infectious Diseases, University Hospital Regensburg, Regensburg, Germany; 4 Institute for Medical Microbiology and Hygiene, University of Regensburg, Regensburg, Germany; 5 Center for Clinical Studies, University Hospital Regensburg, Regensburg, Germany

**Keywords:** COVID-19, SARS-CoV-2, Antibody-mediated immunity, SARS-CoV-2-vaccination, Avidity, Surrogate neutralization

## Abstract

**Background:**

The immune response to COVID-19-vaccination differs between naïve vaccinees and those who were previously infected with SARS-CoV-2. Longitudinal quantitative and qualitative serological differences in these two distinct immunological subgroups in response to vaccination are currently not well studied.

**Methods:**

We investigate a cohort of SARS-CoV-2-naïve and COVID-19-convalescent individuals immediately after vaccination and 6 months later. We use different enzyme-linked immunosorbent assay (ELISA) variants and a surrogate virus neutralization test (sVNT) to measure IgG serum titers, IgA serum reactivity, IgG serum avidity and neutralization capacity by ACE2 receptor competition.

**Results:**

Anti-receptor-binding domain (RBD) antibody titers decline over time in dually vaccinated COVID-19 naïves whereas titers in single dose vaccinated COVID-19 convalescents are higher and more durable. Similarly, antibody avidity is considerably higher among boosted COVID-19 convalescent subjects as compared to dually vaccinated COVID-19-naïve subjects. Furthermore, sera from boosted convalescents inhibited the binding of spike-protein to ACE2 more efficiently than sera from dually vaccinated COVID-19-naïve subjects.

**Conclusions:**

Long-term humoral immunity differs substantially between dually vaccinated SARS-CoV-2-naïve and COVID-19-convalescent individuals. Booster vaccination after COVID-19 induces a more durable humoral immune response in terms of magnitude and quality as compared to two-dose vaccination in a SARS-CoV-2-naïve background.

**Supplementary Information:**

The online version contains supplementary material available at 10.1007/s15010-022-01817-8.

## Introduction

We are currently experiencing yet another wave of the SARS-CoV-2 pandemic worldwide, with rapidly increasing numbers of cases in many countries, caused mainly by non-vaccinated individuals despite broad vaccination campaigns [1–3]. However, it has become evident that also fully (dually) vaccinated individuals can get infected by SARS-CoV-2 and become symptomatically ill, albeit rarely with a severe course of disease [1, 4]. It appears that immunity to SARS-CoV-2 wanes over time after both SARS-CoV-2 infection and dual vaccination, so that earlier expectations that dual vaccination would provide long-term protective immunity to COVID-19 have not been met [5]. The underlying mechanisms for what appears to be a relatively rapid decay of protective immunity to SARS-CoV-2 are as yet unclear.

We previously described the natural course of antibody levels directed against the SARS-CoV-2 receptor-binding domain as well as SARS-CoV-2 reactive interferon-γ producing T cells over a 1-year period in a cohort of 136 hospital employees who developed COVID-19 during the first wave of SARS-CoV-2 infections between March and May 2020 [6, 7]. We also reported the effects of a single dose booster vaccination with the various licensed COVID-19 vaccines on antibody levels in COVID-19 convalescents and in 30 healthy, COVID-19 naïve individuals after dual vaccination [7].

In this follow-up investigation, we describe the further course of antibody titers over a 6-month period after vaccination in the same cohorts and characterize the vaccine-induced humoral immunity in depth by quantification of the serum avidity and ACE2 competitive neutralization capacity.

## Methods

### Study cohort and blood sampling

The cohort of this study has been described in detail previously [6, 7]. In brief, employees of the Kliniken Südostbayern Hospital Network (Bavaria, Germany) who recovered from a RT-PCR-confirmed COVID-19 episode between April and June 2020 were asked to participate in the prospective cohort study. After written informed consent, participants were asked to provide samples (collected in S-Monovette syringes, Sarstedt, Nümbrecht, Germany) during various time points after recovery. When vaccines against COVID-19 had been approved by heath officials and became available for general use, those of the participants who agreed to receive a booster vaccination (according to the recommendations of the German vaccination advisory board (STIKO [8]) were asked to provide serum samples immediately prior to vaccination, and approximately 14 days and 6 months thereafter.

Healthy employees of the Kliniken Südostbayern and the University Hospital Regensburg without evidence of prior COVID-19 according to symptoms, negative anti-SARS-CoV-2 antibodies and repeated consistently negative SARS-CoV-2 PCR-tests served as controls and underwent the standard two-dose vaccine schedule between February and April 2021 in accordance with STIKO recommendations. They were asked to provide a serum sample immediately prior to the second vaccination, a second sample at least 14 days thereafter and a third sample after approximately 6 months. To exclude the possibility of asymptomatic breakthrough-infection, the absence of antibodies specific for SARS-CoV-2’s nucleoprotein (N) in the serum sample taken 6 month after complete vaccination was furthermore analyzed using Roches Elecsys N-Test (data not shown). As a prerequisite of such analysis, the Elecsys N-Test has been reported to be highly specific and sensitive [9].

Serum was obtained from the blood samples by centrifugation within 6 h after drawing the blood and stored at − 20 °C until analysis.

The study was approved by the University of Regensburg ethic committee (reference number 20-1896-101).

### Detection of SARS‑CoV‑2 nucleoprotein‑specific antibodies

Elecsys Anti-SARS-CoV-2 N-Test (Roche Diagnostics GmbH, Penzberg, Germany) was performed on a COBAS pro e 801 module according to the manufacturer’s recommendations and cutoff values were chosen as specified by the manufacturer.

### Detection of SARS‑CoV‑2‑spike‑protein receptor‑binding domain‑specific antibodies by ELISA

Anti-SARS-CoV-2-specific antibody levels in serum were detected by an ELISA utilizing the SARS-CoV-2-spike protein’s receptor-binding domain (RBD) as antigen, as previously described [10]. The assay is able to detect IgM-, IgA- and IgG-SARS-CoV-2 antibody responses separately with high specificity and sensitivity and the detected antibody levels were shown to correlate well with the virus neutralization capacity of the respective serum sample [9]. The IgG antibody levels in serum samples after vaccination and booster vaccination, respectively, were titrated in eight steps of twofold dilutions, starting at a dilution of 1:200. Endpoint titers were calculated by least squares regression of the individual titration-data using a four parameter logistic curve. A predetermined assay-specific cutoff value was subsequently used together with the parameters from the curve fit, to determine the corresponding endpoint titer dilution. IgA serum reactivities were measured in 1:100 serum dilutions and are given in signal-to-cutoff ratios as described earlier [10].

### Analysis of the serum avidity

To determine the serum avidity [11], the previously described ELISA [10] was modified as follows (all reagents were used as described before). Sera were titrated in eight 2.5-fold serial dilutions starting at 1:40 dilution in two side by side replicates. After the serum binding step, the wells were washed ten times with 200 µl phosphate buffered saline (PBS), containing 0.1% Tween 20 (PBS-T). Thereupon, one replicate was treated with 100 µl of 1.5 M sodium thiocyanate (NaSCN) in PBS per well while the other replicate was treated with PBS. After 15 min incubation at ambient temperature, the plate was washed again with PBS-T, conjugate was added and the ELISA was continued as previously described. The calculation of the avidity index is described below (see “Data analysis and statistics”).

### ACE2-NanoLuc surrogate virus neutralization assay (sVNT)

ELISA formats to determine SARS-CoV-2 neutralizing antibodies that compete with ACE2-receptor binding have been described to correlate well with virus neutralization [13–15]. In principle, the S-protein or its RBD is immobilized on a solid phase, incubated with serum, ACE2 is added and its binding in comparison to a non-serum-bound control is quantified. Our in-house sVNT uses an ACE2 variant that is N-terminally fused to the *Oplophorus gracilirostris* luciferin 2-monooxygenase (NanoLuc [16]). The construct, which provides a C-terminal octahistidine purification tag, was optimized for human codon usage, synthesized by GeneArt AG (Thermo Fisher Scientific) and cloned into a pcDNA3.1 mammalian expression vector. Expression was performed in Expi293F cells according to the manufacturer’s recommendations. After 5 days of protein expression, supernatants were loaded onto an immobilized metal chelate affinity chromatography (IMAC) column (HisTrap Excel, Cytiva), washed with PBS (Sigma) containing 10 mM imidazole (Sigma) and eluted by a linear gradient of 10–500 mM imidazole in PBS. After buffer exchange to 10 mM NaCl in HEPES pH 6.8 the protein was further purified by anion exchange chromatography (HiTrap DEAE Sepharose, Cytiva) using a gradient from 10 mM to 1 M NaCl, in HEPES pH 6.8.

For the competitive ELISA, sera were diluted 1:50 in 1% fat free milk in PBS (Gibco) supplemented with 0.1% Tween 20 (Caelo) (PBS-T) and added to RBD-coated (1 µg/ml over night at 4 °C) and pre-blocked (5% at free milk in PBS) ELISA plate (LumiNunc 96-well plate, Thermo Scientific). After 1 h, the plate was washed with PBS-T and 200 nM NanoLuc-ACE2 in PBS-T was added for 30 min. After washing with PBS-T, 50 µl Nano-Glo Luciferase Assay Reagent (Promega) was added to each well and the luminescence signal was detected within 20 min in a 96 well luminescence reader (VICTOR Plate Reader, PerkinElmer). The luminescence counts per second were normalized to the signal of a control well without serum competition and to the median signal from all SARS CoV-2 naïve sera.

### Data analysis and statistics

To determine the avidity index [12], the OD_450–630 nm_ values were analyzed by a least squares regression fitting using a four parameter logistic curve. Area under the curve (AUC) was calculated (GraphPad Prism for Windows 9.0; GraphPad, San Diego/USA) and the ratio of the AUC with and without NaSCN was calculated as avidity index (AI) according to Eq. 1.

1 $$\mathrm{AI }= \frac{{\mathrm{AUC }}_{\mathrm{NaSCN}}}{{\mathrm{AUC}}_{\mathrm{ PBS}}}.$$

Descriptive statistics were calculated from raw data using SPSS (SPSS Statistics 26, IBM, New York/USA) and GraphPad Prism (GraphPad Prism for Windows 9.0; GraphPad, San Diego/USA). Kruskal–Wallis test was used for nonparametric comparison of groups, with Dunn's test of multiple comparisons post hoc to correct for multiple testing. Graphs were generated with Graphpad Prism.

## Results

### Anti-SARS‑CoV‑2‑spike‑protein receptor‑binding domain‑specific antibody levels

**Fig. 1:**
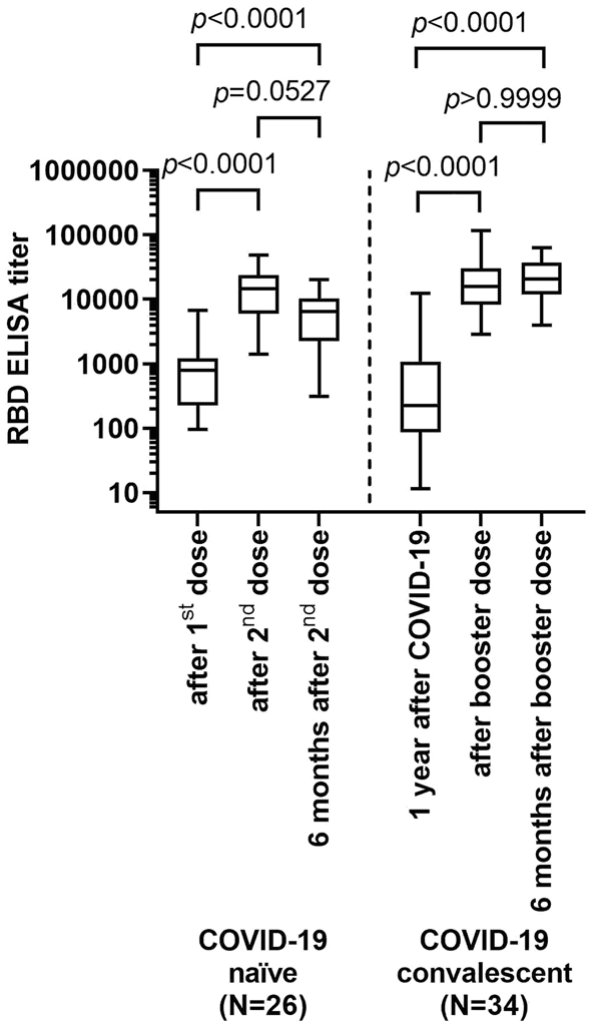
Anti-spike protein receptor-binding domain (RBD) IgG antibody titers at different time points relative to vaccinations and booster doses in COVID-19-naïve individuals and COVID-19 convalescents (Kruskal–Wallis test)

The 26 COVID-19-naïve individuals in the control group experienced a considerable increase in anti-RBD IgG titer from before 2nd vaccine dose to after 2nd vaccine dose (Fig. 1, median anti-RBD-titers 794 vs. 14,524, respectively, *p* < 0.0001). Over a 6-month period after the 2nd vaccine dose (median 150 days, IQR 142–196 days), anti-RBD-titers decreased again significantly, but still remained higher than after the 1st vaccine dose (median anti-RBD titer 6500, *p* = 0.0527) compared to after 2nd dose. Same applies to the anti-RBD IgA reactivity (Fig. S1).

For 34 COVID-19 convalescents, blood samples were available prior to administration of the booster dose, almost one year after infection (median 326 days, inter-quartile rage (IQR) 171–337 days). By then, median anti-RBD IgG titers were below median titers of the single vaccinated naïve persons (Fig. 1). After booster vaccination, anti-RBD-titers increased significantly (median anti-RBD-titers 14,610 vs. 226, respectively; *p* < 0.0001), with levels similar to those in COVID-19-naïve individuals after the second vaccine dose. In contrast to COVID-19-naïve individuals, anti-RBD-titers did not decrease over the 6-month period (median 169 days, IQR 142–180 days) thereafter (median anti-RBD-titers 14,610 and 20,594, respectively; *p* > 0.9999). No significant differences in anti-RBD-titers were noted between individuals vaccinated with ChAdOx1-S (Oxford/Astra-Zeneca; *N* = 11), BNT162b2 (Pfizer/Biontech; *N* = 10), and mRNA-1273 (Moderna; *N* = 13), respectively, both immediately after vaccination and 6 months thereafter (Fig. S2a). RBD directed IgA-serum-reactivities were higher in COVID-19 convalescents as compared to SARS-CoV-2 naïve subjects immediately after vaccination (*p* = 0.0158) and 6 months after vaccination (*p* < 0.0001, Fig. S1).

### Serum avidity

**Fig. 2:**
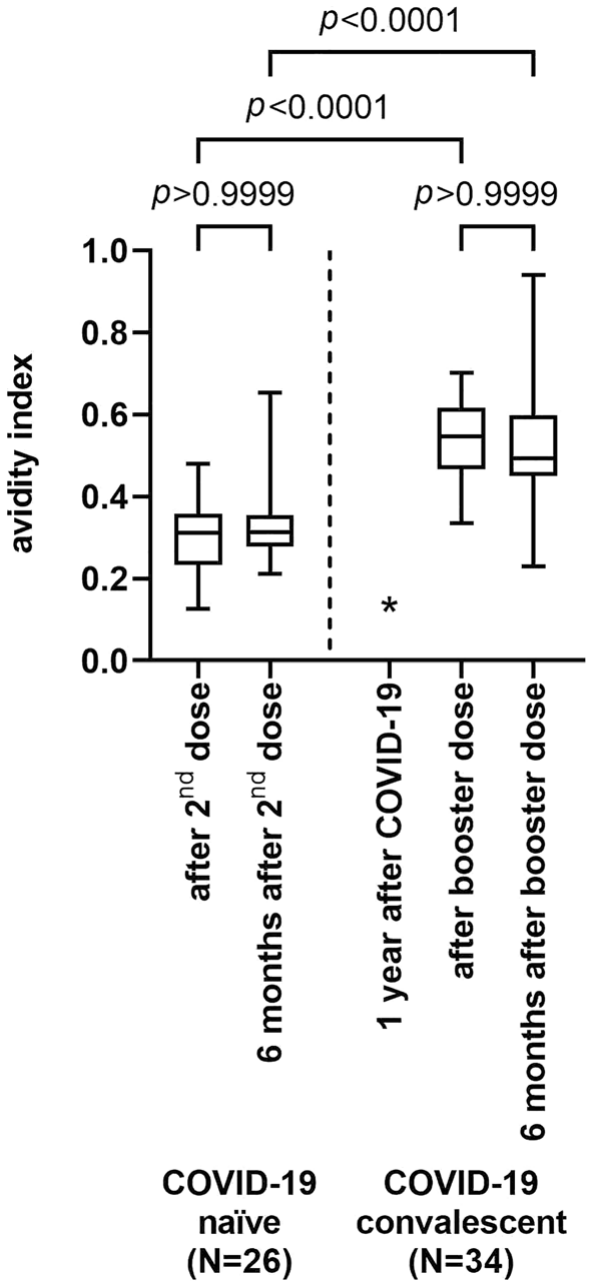
Antibody avidity index of COVID-19-naïve subjects after vaccination and after 6 months of follow-up, and of COVID-19 convalescents after booster vaccination and after 6 months of follow-up (Kruskal–Wallis test). Data from avidity testing of sera from convalescents prior to vaccination gave low signals overall and avidity indices could not be determined (*)

The avidity of anti-RBD IgG serum antibodies in COVID-19 convalescents after booster vaccination proved considerably higher than the avidity of serum of the dually vaccinated uninfected control group (Fig. 2, median avidity index 0.5455, IQR 0.4641–0.6139 vs. 0.3117, IQR 0.2326–03,576; *p* < 0.0001). Over the 6 month period thereafter, the avidity index of anti-RBD-antibodies in dually vaccinated COVID-19 naïves remained unchanged (median avidity index 0.3135, IQR 0.2789–0.3552), and decreased slightly numerically but not significantly in boosted COVID-19 convalescents (median avidity index 0.3145, IQR 0.2811–0.3538; *p* < 0.0001 for comparison between dually vaccinated naïves and boosted convalescents after 6 months). Analysis of anti-RBD antibody avidity in COVID-19 convalescents prior to booster vaccination was not possible, due to the overall low antibody levels. Of note, anti-RBD binding avidity and anti-RBD titer as well as anti-RBD binding avidity and ACE2 receptor binding inhibition did not correlate (Fig. S3). No significant differences in anti-RBD-affinity were noted between individuals vaccinated with ChAdOx1-S, BNT162b2 and mRNA-1273, respectively, both immediately after vaccination and 6 months thereafter (Fig. S2 b).

### ACE2-receptor antibody binding competition assay

**Fig. 3:**
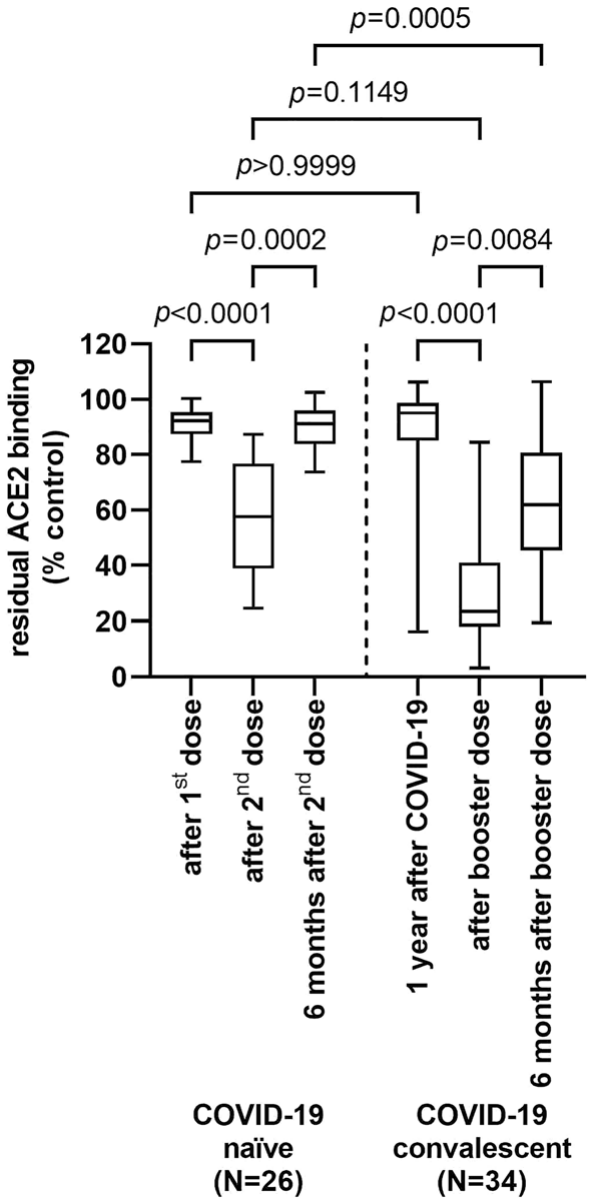
Competitive ACE2-receptor binding inhibition by sera of COVID-19 naïve controls after first vaccine dose, after second vaccine dose and after 6 months of follow-up, and of COVID-19 convalescents prior to booster vaccination, after booster vaccination and after 6 months of follow-up (Kruskal–Wallis test)

**Fig. 4:**
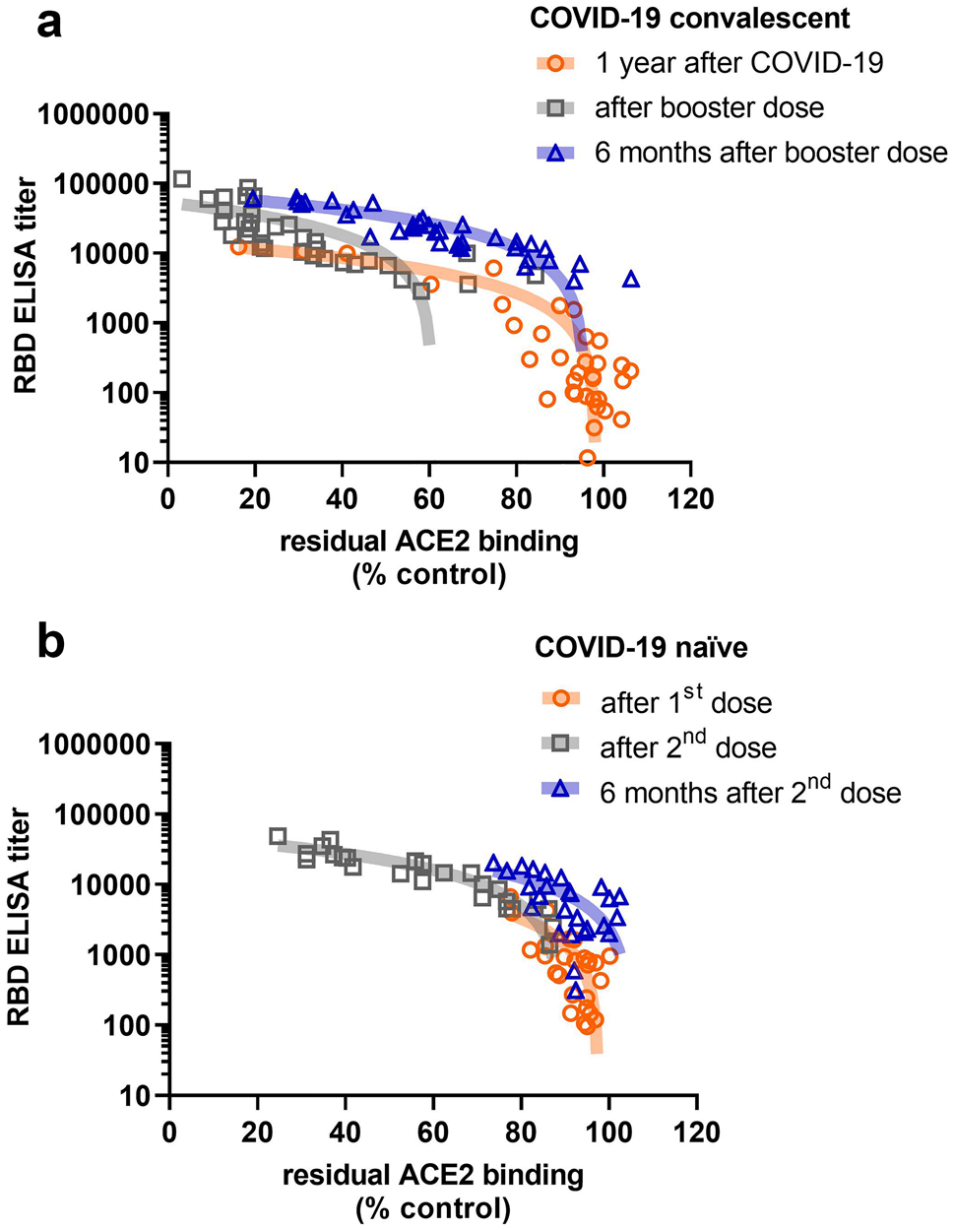
Correlation of antibody binding titer and ACE2-receptor binding inhibition over time among COVID-19-naïve, dually vaccinated individuals (**a**) and COVID-19 convalescents after a single booster dose (**b**) (data and linear regression line displayed). Associated Pearson r/Spearman's rho values are – 0.9463/− 0.6908 for COVID-19 convalescents 1 year after COVID-19, − 0.6245/− 0.8915 for COVID-19 convalescents after booster dose, − 0.9033/− 0.9224 for COVID-19 convalescents 6 months after booster dose, − 0.7255/− 0.5911 for COVID-19-naïves after the 1st vaccine dose, − 0.8958/− 0.9556 for COVID-19-naïves after the 2nd vaccine dose, and − 0.6900/− 0.6226 for COVID-19-naïves 6 months after the 2nd vaccine dose (all corresponding two-tailed *p* values of the correlations were *p* < 0.0001)

Inhibition of spike-protein binding to the ACE2 receptor was significantly increased by sera of vaccinated COVID-19 naïve individuals immediately after the second vaccine dose (Fig. 3, median residual binding, 92% vs. 58%, *p* < 0.0001), but returned to the levels observed after vaccination with the first vaccine dose after 6 months (median residual binding, 91%). Prior to booster vaccination, COVID-19 convalescents showed similar levels of ACE2 receptor binding inhibition as COVID-19 naïves after the first vaccine dose or 6 months after the second dose (median residual binding, 95%). However after the booster dose, ACE2 receptor binding inhibition by sera from COVID-19 convalescents was by trend, but not significantly enhanced compared to dually vaccinated COVID-19 naïves (median residual binding 23% vs. 58%, *p* < 0.1149). The inhibitory property was much better retained over 6 months in boosted COVID-19 convalescents than in dually vaccinated COVID-19 naïves (median residual binding 62% vs. 91%, *p* = 0.0005) (Fig. 3). Anti-RBD-antibody titer and ACE2 binding inhibitory properties are significantly correlated (Fig. 4). Both COVID-19 naïve controls and COVID-19 convalescents show a shift towards higher antibody titers and more binding inhibition after the second vaccine dose and the booster dose, respectively (Fig. 1); however after 6 months ACE2 binding inhibition almost completely disappeared despite still elevated antibody titers in dually vaccinated COVID-19 naïve subjects, whereas among boosted convalescents ACE2 binding inhibition was much better preserved (Fig. 3).

No significant differences in ACE2 binding inhibition were noted between individuals vaccinated with ChAdOx1-S, BNT162b2 and mRNA-1273, respectively, both immediately after vaccination and 6 months thereafter (Fig. S2c).

## Discussion

Several investigations have analyzed levels of vaccine- or infection-induced anti-SARS-CoV-2 antibody levels over time and the protective potential of high antibody titers has been demonstrated [17–19]. However, antibody levels that reliably predict protective immunity against COVID-19 have not been defined so far. In this study we sought to characterize the immune response against the SARS-CoV-2 spike protein in dually vaccinated COVID-19 naïve subjects and boosted COVID-19 convalescent subjects not only on the basis of anti-spike-antibody levels, but by additional tests that give estimates of antibody "quality". Specifically, we analyzed anti-RBD-antibody avidity and used a functional ACE2 binding competition assay to quantify receptor-competition-based neutralization capacity of the sera.

In accordance with other studies we found declining anti-RBD IgG antibody titers and IgA serum reactivity over time in dually vaccinated COVID-19 naïve persons [20], whereas the titers in boosted COVID-19 convalescents are higher and more stable [21–23].The two additional antibody attributes evaluated here describe further differences between dually vaccinated COVID-19 naïve individuals and boosted COVID-19 convalescents.

Only few investigations have so far addressed the avidity of anti-SARS-CoV-2 antibodies. In this study, the antibody avidity proved to be considerably higher among boosted COVID-19 convalescents than among dually vaccinated COVID-19 naïves. This is in line with the findings by Tang et al*.* [24], who also described lower binding of post-vaccination sera from naïve compared to convalescent individuals. In contrast to an expected increase in avidity over time [25], which was observed in longitudinal analyses of the immune response against SARS-CoV-2 after COVID-19 [26], the avidity index in this study remained unchanged after 6 months (in boosted convalescents). However, as already 1 year had elapsed after the primary COVID-19 infection when the patients in this study were vaccinated it could well be, that affinity maturation had already been completed in most individuals [26]. Expectedly, we were unable to show an association between anti-RBD-antibody avidity and titers, suggesting that the kinetics of B cell maturation, plasma cell expansion and antibody production are different.

Similarly, sera from boosted convalescents inhibited spike-protein to ACE2 receptor binding more effectively than sera from dually vaccinated COVID-19 naïves, and this activity persisted better over time in boosted convalescents than in dually vaccinated COVID-19 naïves. Despite correlation between antibody titers and the ACE2 binding inhibiting activity, this more functional competitive inhibition assay therefore appears to describe yet another quality of the humoral response against the SARS-CoV-2 virus.

While this study was neither designed nor powered to assess the impact of these immunological findings on actual protection from COVID-19 reinfection, these observations provide a good explanation for the better protection from COVID-19 reinfection of boosted convalescents compared to dually vaccinated COVID-19 naïve individuals observed in large epidemiological studies [27, 28]. According to current understanding, a longer, broader and more intense interaction of T-follicular helper cells in infected and subsequently boosted individuals is thought to underlie this phenomenon [29, 30]. It will be interesting to see how a third vaccine dose may shape the immune response in COVID-19 naïves. Non-boosted convalescents appear to be less protected from COVID-19 reinfection than dually vaccinated COVID-19 naïves according to large cohort analyses [31], which again compares very well with our results, as the respective subcohort in this study also showed low antibody titers and little ACE2 binding inhibition.

As a limitation of this study, the analyses describe only aspects of the humoral response; cell-mediated immunity will certainly also play a prominent role in the immune defense against SARS-CoV-2 and remains to be studied after vaccination in COVID-19 naïve persons compared to convalescents. However, a strength of this study is the extended follow-up of this cohort over at least 18 months with detailed serological analysis in a considerable number of individuals who provided serum samples in all the relevant time periods.

In conclusion, this study shows considerable differences in the long-term humoral immunity between dually vaccinated COVID-19 naïve and COVID-19 convalescent individuals. It appears that a booster vaccination after natural COVID-19 infection provides a more sustained humoral immune response in terms of magnitude (titers) and quality (avidity and surrogate neutralization capacity) than vaccination with two COVID-19 vaccine doses, fully congruent with clinical epidemiological observations.

## Supplementary Information

Below is the link to the electronic supplementary material.

Supplementary file1 (PDF 565 kb)
