# Supplementary material for: Humoral immunity in dually vaccinated SARS-CoV-2-naïve individuals and in booster-vaccinated COVID-19-convalescent subjects
Source: Infection. 2022 Apr 11;50(6):1475–81. doi: 10.1007/s15010-022-01817-8 (PMC8995884; doi:10.1007/s15010-022-01817-8)
Supplement: Supplementary file 1 — Supplementary file1 (PDF 565 kb) [file 15010_2022_1817_MOESM1_ESM.pdf]

Online Supplementary Material

Humoral Immunity in Dually Vaccinated SARS-CoV-2 Naïve Individuals and in Booster-Vaccinated COVID-19 Convalescent Subjects

Vivian Glück[1], Leonid Tydykov[1], Anna-Lena Mader[1], Anne-Sophie Warda[2], Manuela Bertok[2], Tanja Weidlich[2], Christine Gottwald[2], Josef Köstler[1], Bernd Salzberger[3], Ralf Wagner[1,4], Michael Koller[5], André Gessner[1,4], Barbara Schmidt[1,4], Thomas Glück[2], David Peterhoff[1,4]*

[1] Institute for Clinical Microbiology and Hygiene, University Hospital Regensburg, Regensburg, Germany

[2] Kliniken Südostbayern AG, Klinikum Traunstein, Traunstein, Germany

[3] Department for Infection Control and Infectious Diseases, University Hospital Regensburg, Regensburg, Germany

[4] Institute for Medical Microbiology and Hygiene, University of Regensburg, Regensburg, Germany

[5] Center for Clinical Studies, University Hospital Regensburg, Regensburg, Germany

* Correspondence: david.peterhoff@ur.de

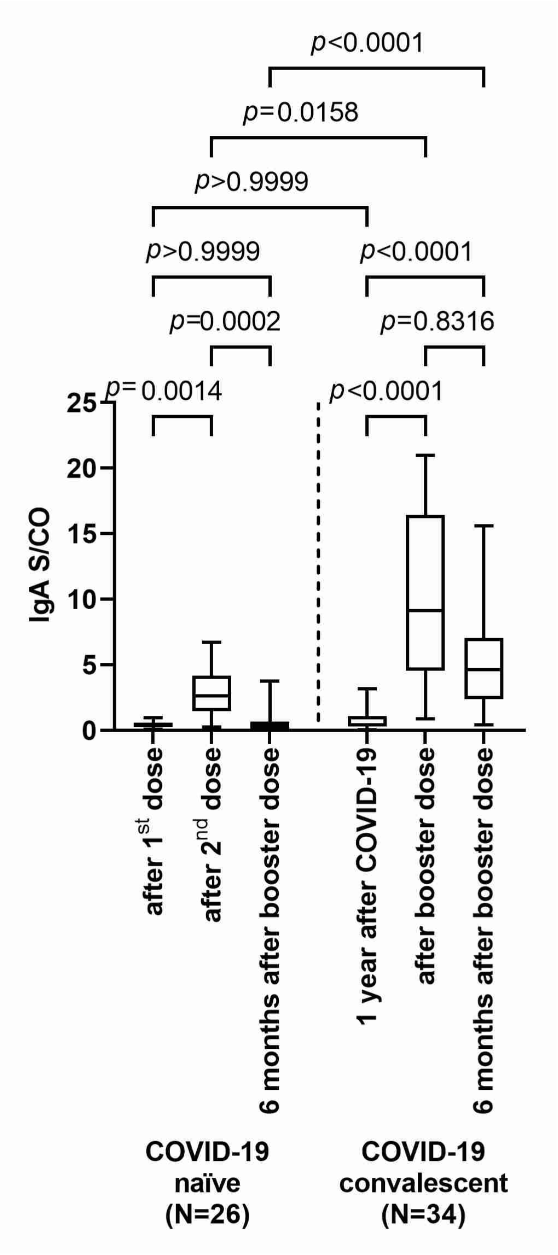

Fig. S1: Anti-spike protein receptor-binding domain (RBD) IgA antibody reactivities (S/CO=signal/cutoff) at different time points relative to vaccinations and booster doses in COVID-19 naïve individuals and COVID-19 convalescent subjects (Kruskal-Wallis-Test with Dunn's multiple comparison post test).

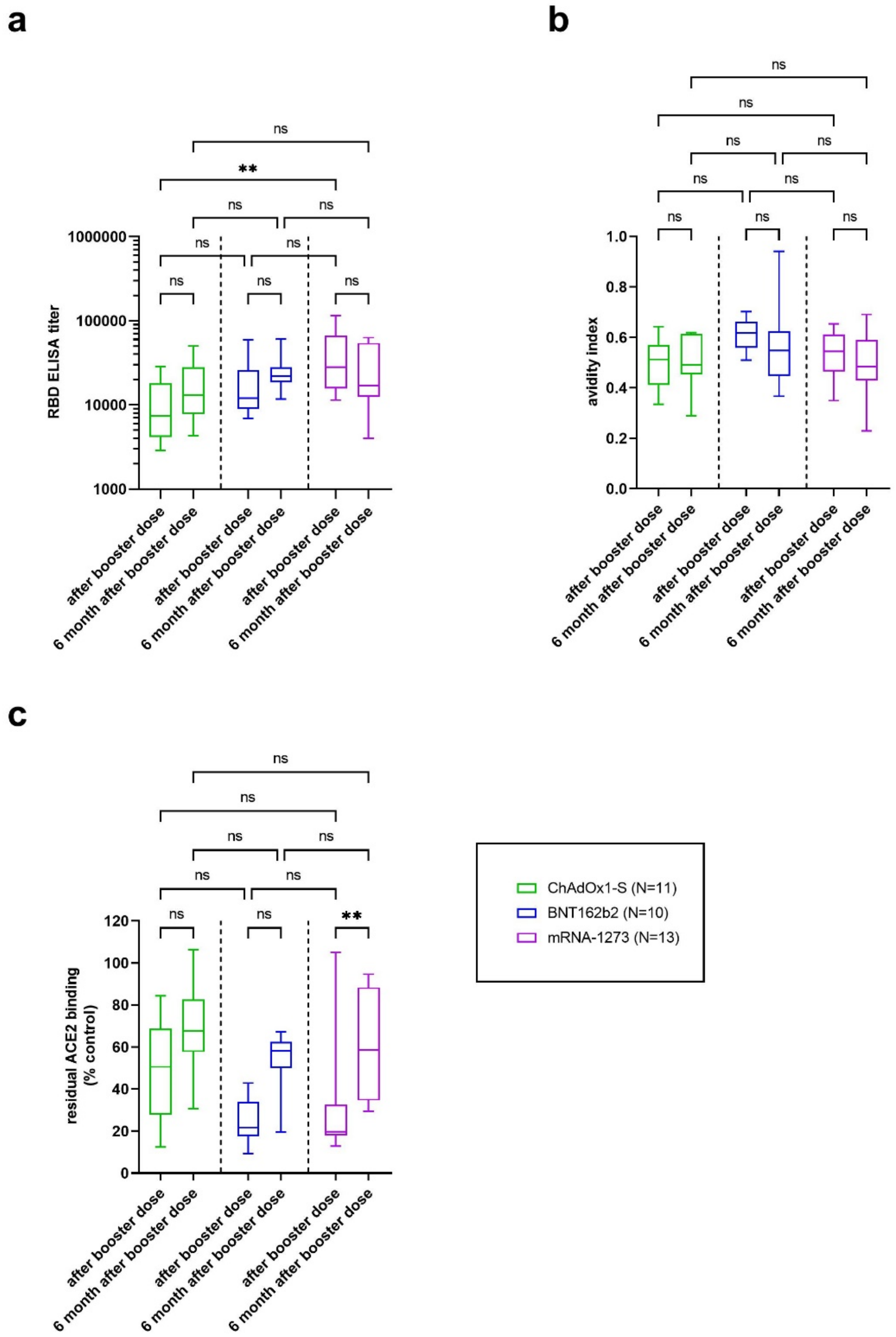

Fig. S2: Anti-spike protein receptor-binding domain (RBD) antibody titers (a), serum avidity index (b), and residual ACE-2 binding (c) at various time points relative to vaccinations and booster doses in COVID-19 naïve individuals and COVID-19 convalescents, stratified according to type of vaccine (Kruskal-Wallis-Test with Dunn's multiple comparison post test, *p*-value style: 0.1234 (ns), 0.0332 (*), 0.0021 (**), 0.0002 (***), <0.0001 (****)).

a

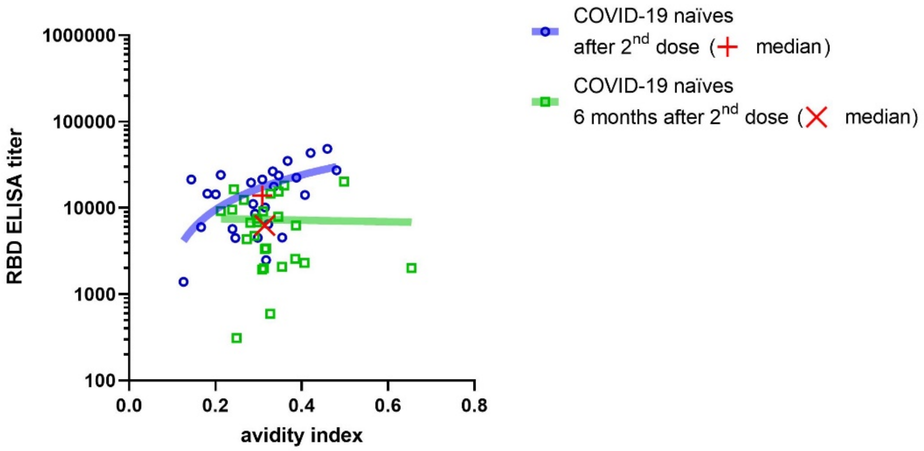

b

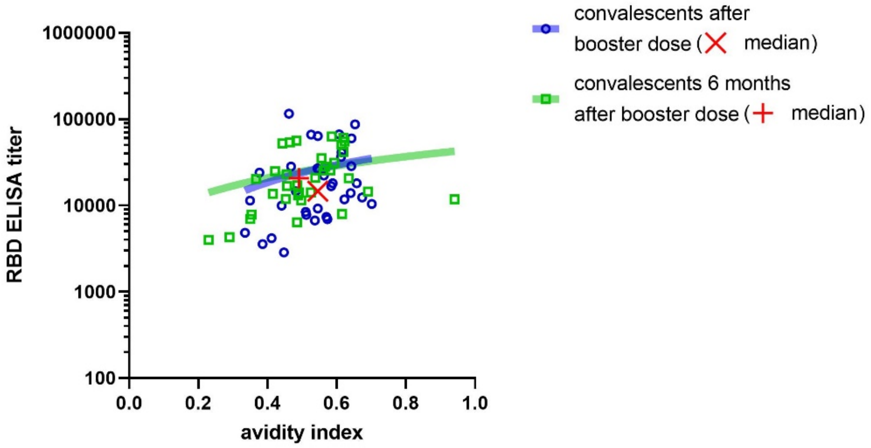

Fig. S3: Correlation between anti-spike protein receptor-binding domain (RBD) antibody titers and serum avidity index at various time points relative to vaccinations and booster doses in COVID-19 naïve individuals (a) and COVID-19 convalescents (b). Associated Pearson r/Spearman's rho values (and their corresponding two-tailed *p*-values) are 0.5469/0.4940 (0.0038/0.0103) for COVID-19-naïves after the 2nd vaccine dose, -0.02277/-0.1029 (0.9121/0.6169) for COVID-19-naïves 6 months after the 2nd vaccine dose, 0.2003/0.3711 (0.2560/0.0307) for COVID-19 convalescents after the booster dose, and 0.2857/0.4203 (0.1014/0.0133) for COVID-19 convalescents 6 months after the booster dose.
